# Supplementary figures and images for: Neurally-constrained modeling of human gaze strategies in a change blindness task
Source: PLoS Comput Biol. 2021 Aug 24;17(8):e1009322. doi: 10.1371/journal.pcbi.1009322 (PMC8478260; doi:10.1371/journal.pcbi.1009322)

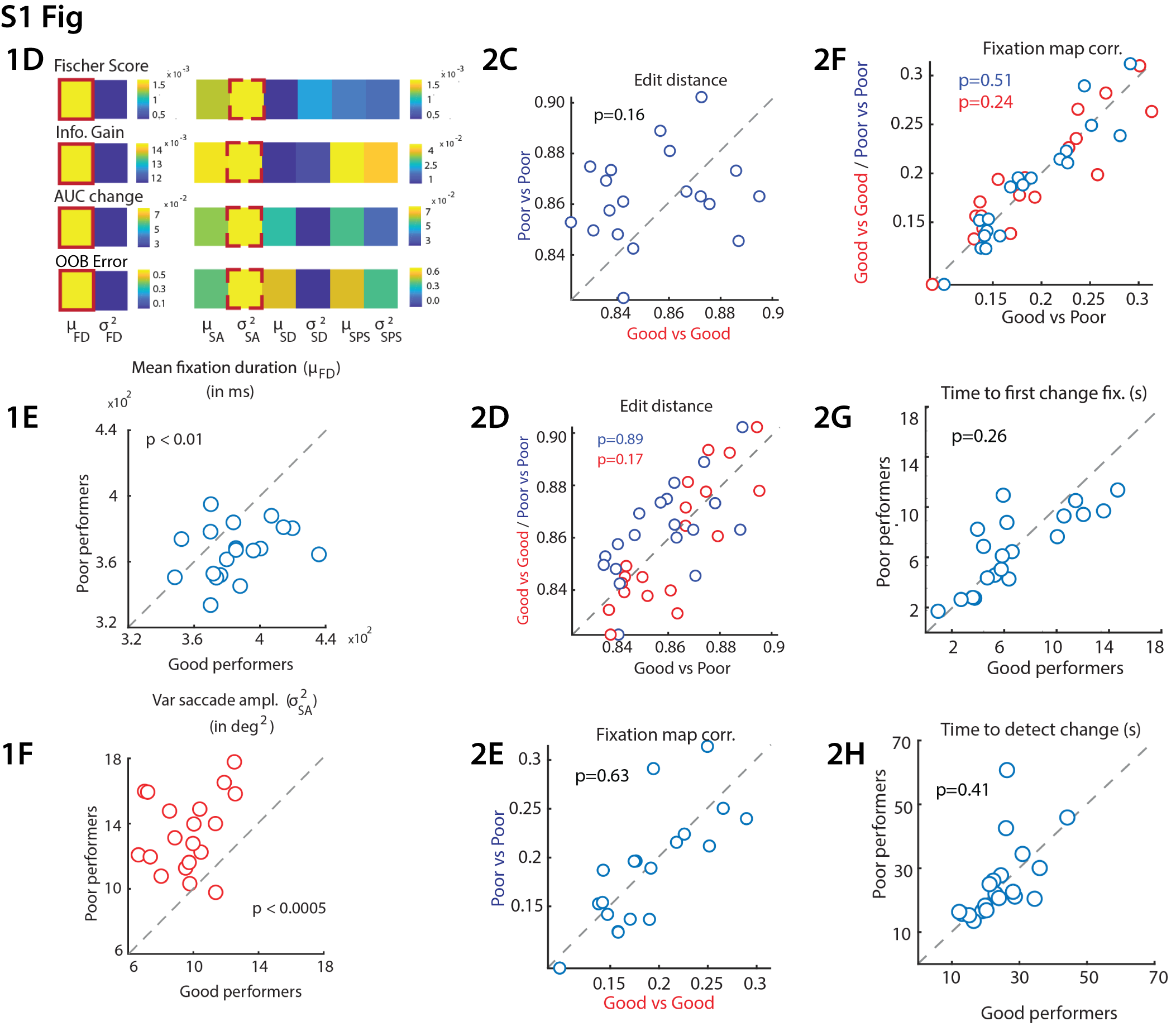

Supplement: S1 Fig — From top to bottom row: Re-analysis of the data shown in Figs 1 and 2 (main text), except that “good” and “poor” performers were defined based on a median split of the data. Other conventions are the same as in the corresponding figure panels in the main text. (TIF) [file pcbi.1009322.s001.tif]

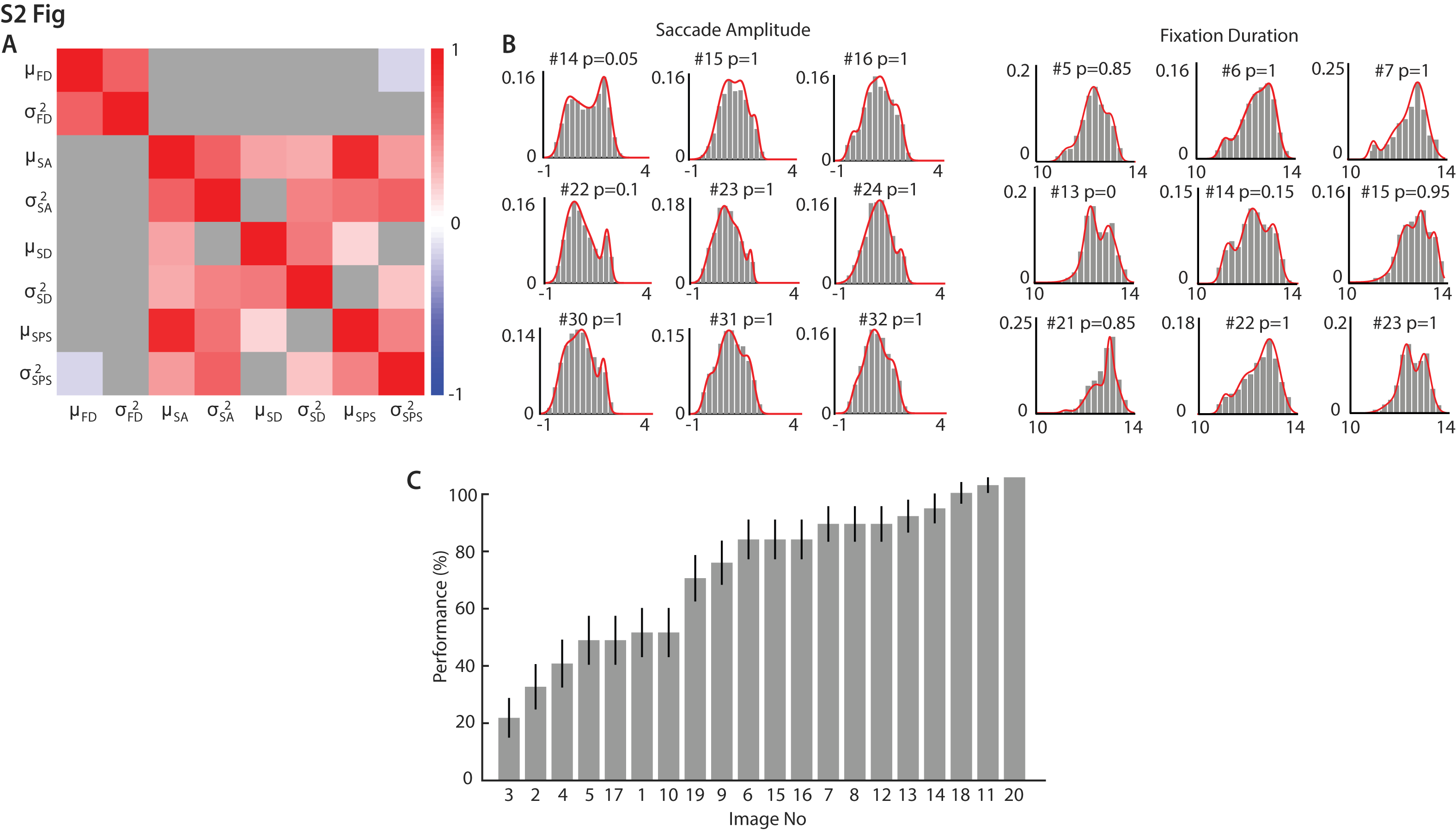

Supplement: S2 Fig — A. Pair-wise correlations among the eight gaze metrics used as features in classification analysis of good versus poor performers (Fig 1C, main text). Gray squares: non-significant correlations. Colored square: significant correlations at p<0.01 with Bonferroni correction for multiple comparisons. Abbreviations are as in Fig 1C (main text). B. Saccade amplitude (left) and fixation duration (right) distributions for representative participants (ID-s in each subplot title). Red fits: Mixture of Gaussians model. p-value in title of each subplot indicates significance level for deviation from unimodality per Hartigan’s dip test (smaller p-values represent greater evidence of bi/multi-modailty). C. Success rates of human observers on the change blindness trial images (n = 20), sorted by the proportion of hits. Error bars denote standard error of the mean performance across participants. (TIF) [file pcbi.1009322.s002.tif]

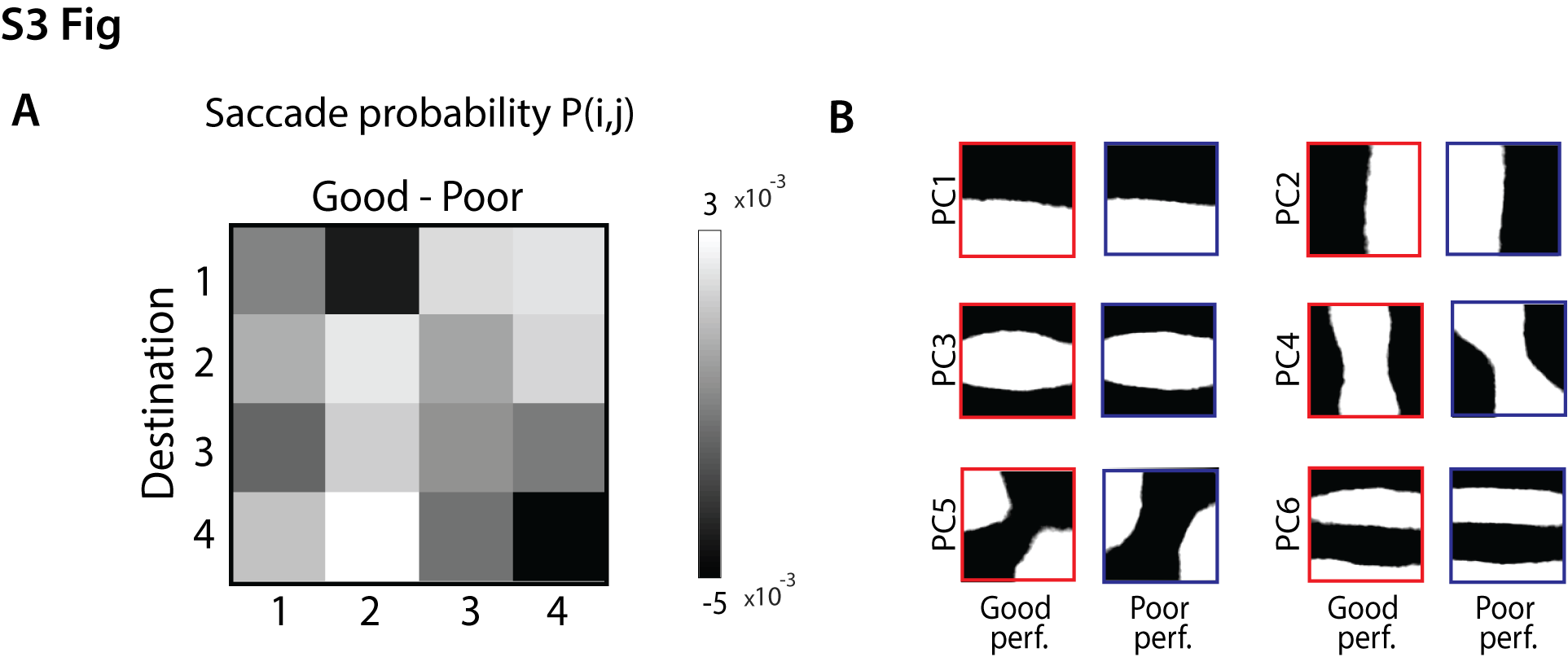

Supplement: S3 Fig — A. Difference between the average saccade probability matrices for the good and poor performers (good minus poor). Other conventions are the same as in Fig 3A (main text). Note that these differences are 3 orders of magnitude smaller than the values in Fig 3A (main text). B. Same as in Fig 3D (main text) except that fixated features were identified following PCA on 112x112 patches extracted from a saliency map, rather than the grayscale image. The saliency map was generated with the frequency tuned saliency algorithm [24]. Other conventions are the same as in Fig 3D main text. (TIF) [file pcbi.1009322.s003.tif]

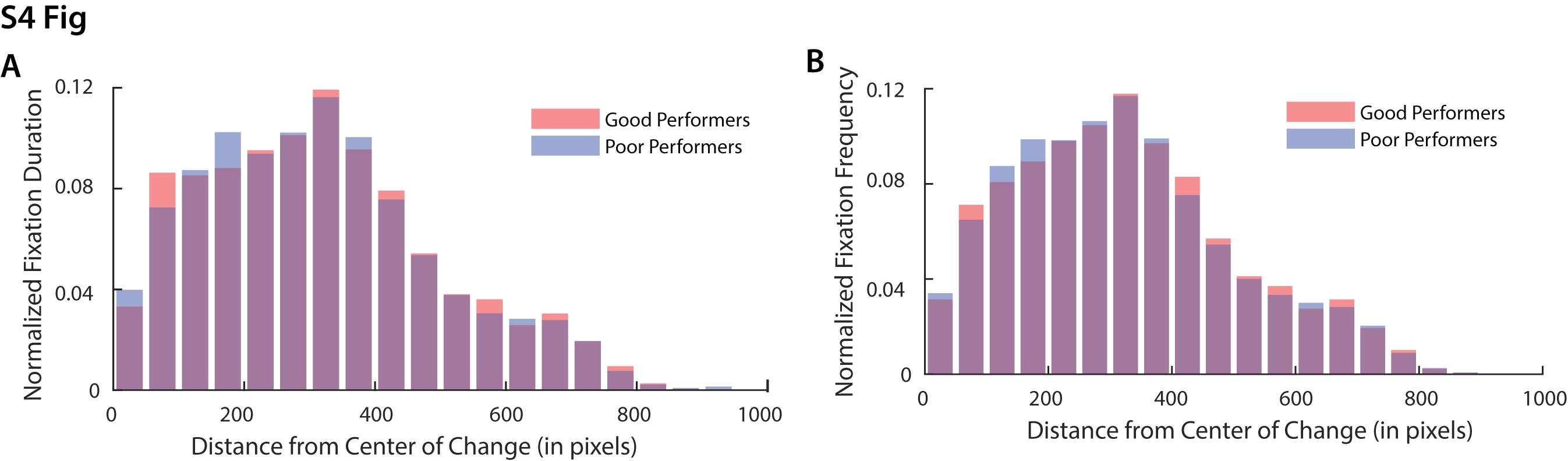

Supplement: S4 Fig — A. Distribution of frequency of fixations, binned based on the distance of fixation relative to the center of the change location, separately for good (red) and poor (blue) performers. B. Same as in panel A but for the total fixation duration. (TIF) [file pcbi.1009322.s004.tif]

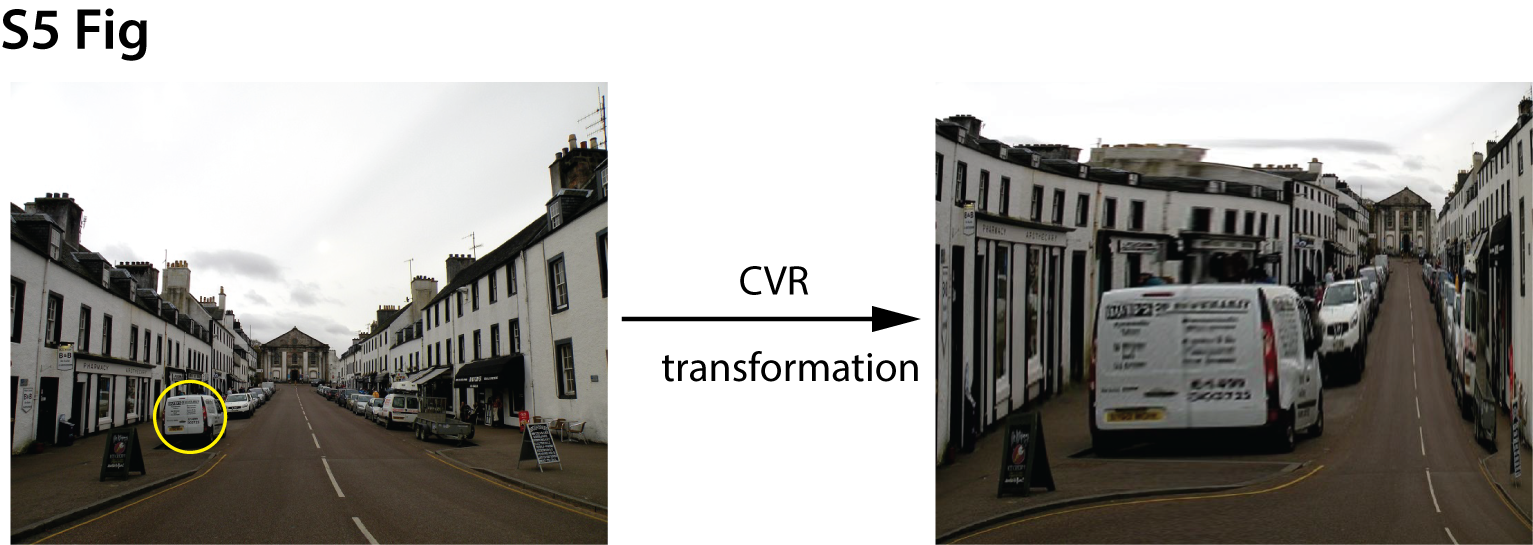

Supplement: S5 Fig — Illustration of foveal magnification with the Cartesian Variable Resolution (CVR) transform for a hypothetical fixation (highlighted by the circle) on one of the images used in the change blindness task (Image #6, S1 Table). (TIF) [file pcbi.1009322.s005.tif]

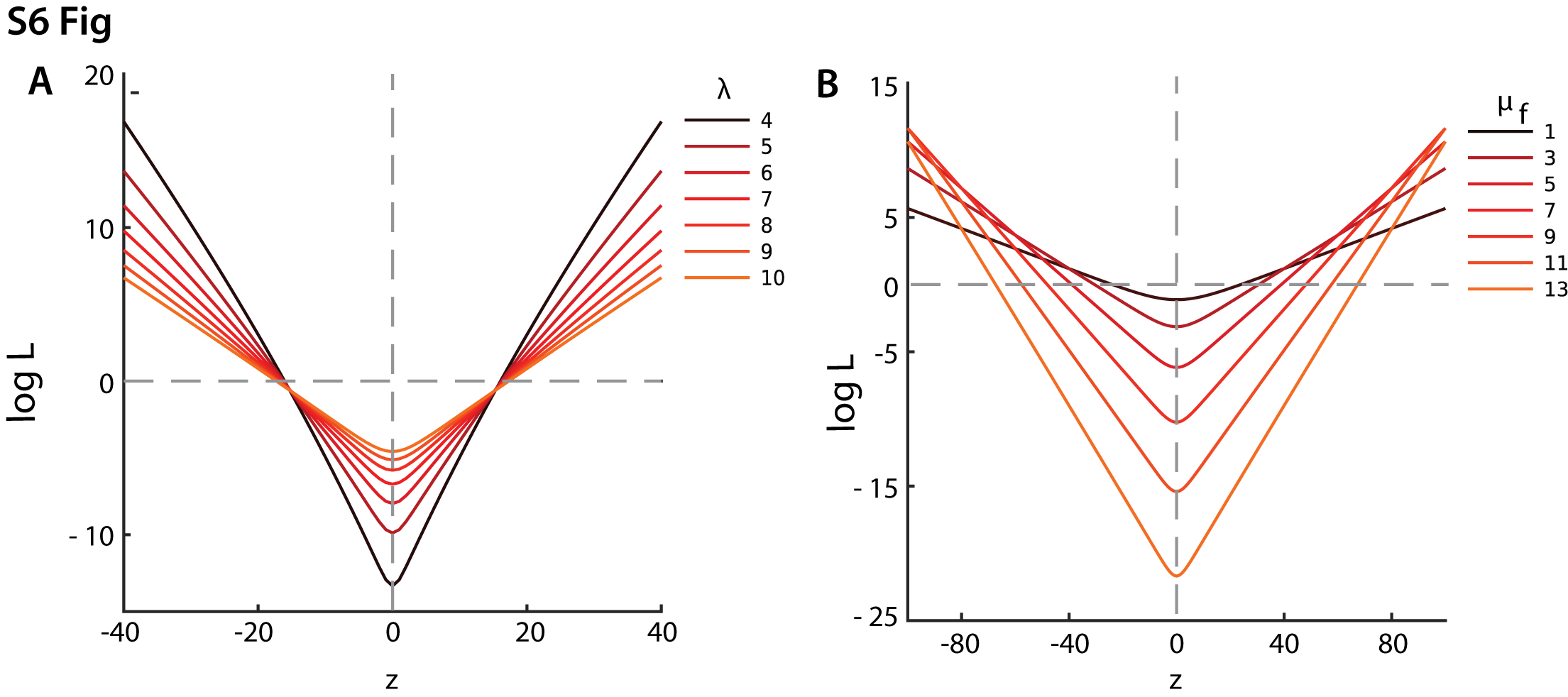

Supplement: S6 Fig — A. Likelihood ratio (L(t; z)) as a function of spike count difference between the first and second image (z, Eq 1; main text) for different values of the mean firing rate, λ = 4 … 10 spikes/bin. The number of time bins for which the first and second images were fixated (m and n−p, respectively) have each been fixed to 5 bins, and the firing rate difference prior, μf fixed at 3 spikes/bin. Curves of progressively lighter shades: increasing values of the mean firing rate. B. Same as in A, but for different values of the firing rate difference prior, μf = 1, 3, 5 … 13 spikes/bin and mean firing rate λ fixed at 40 spikes/bin. Curves of progressively lighter shades: increasing values of μf. (TIF) [file pcbi.1009322.s006.tif]

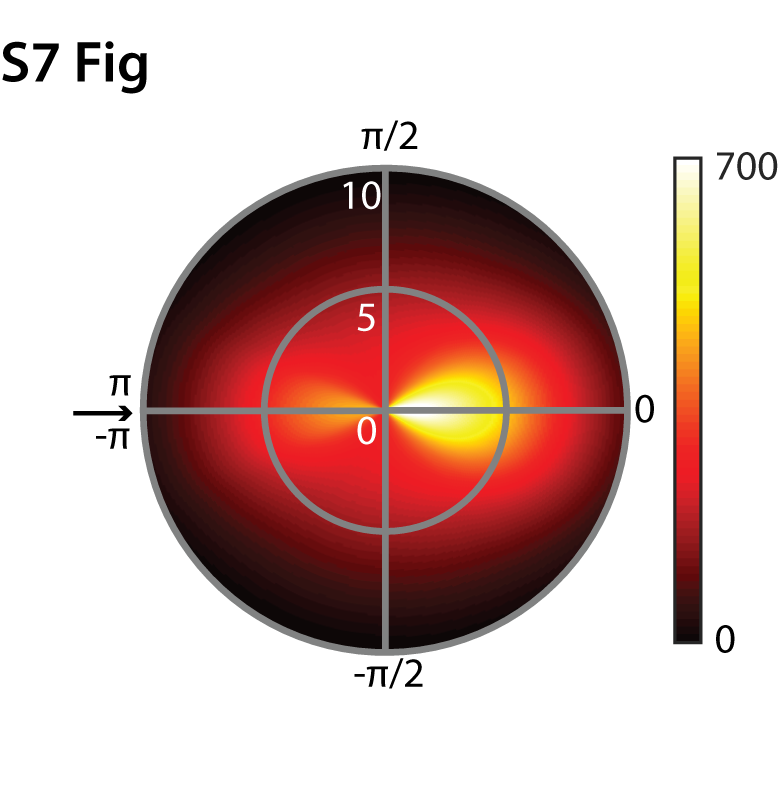

Supplement: S7 Fig — Polar heat map indicating the distribution of human saccade amplitudes and turn angles. The arrow indicates the location of the last saccade. The histogram was computed using data from all (n = 39) participants and all (n = 20) images. The bias against right angled turns is apparent. The distribution was smoothed both along the radial and angular directions, for display purposes only. (TIF) [file pcbi.1009322.s007.tif]

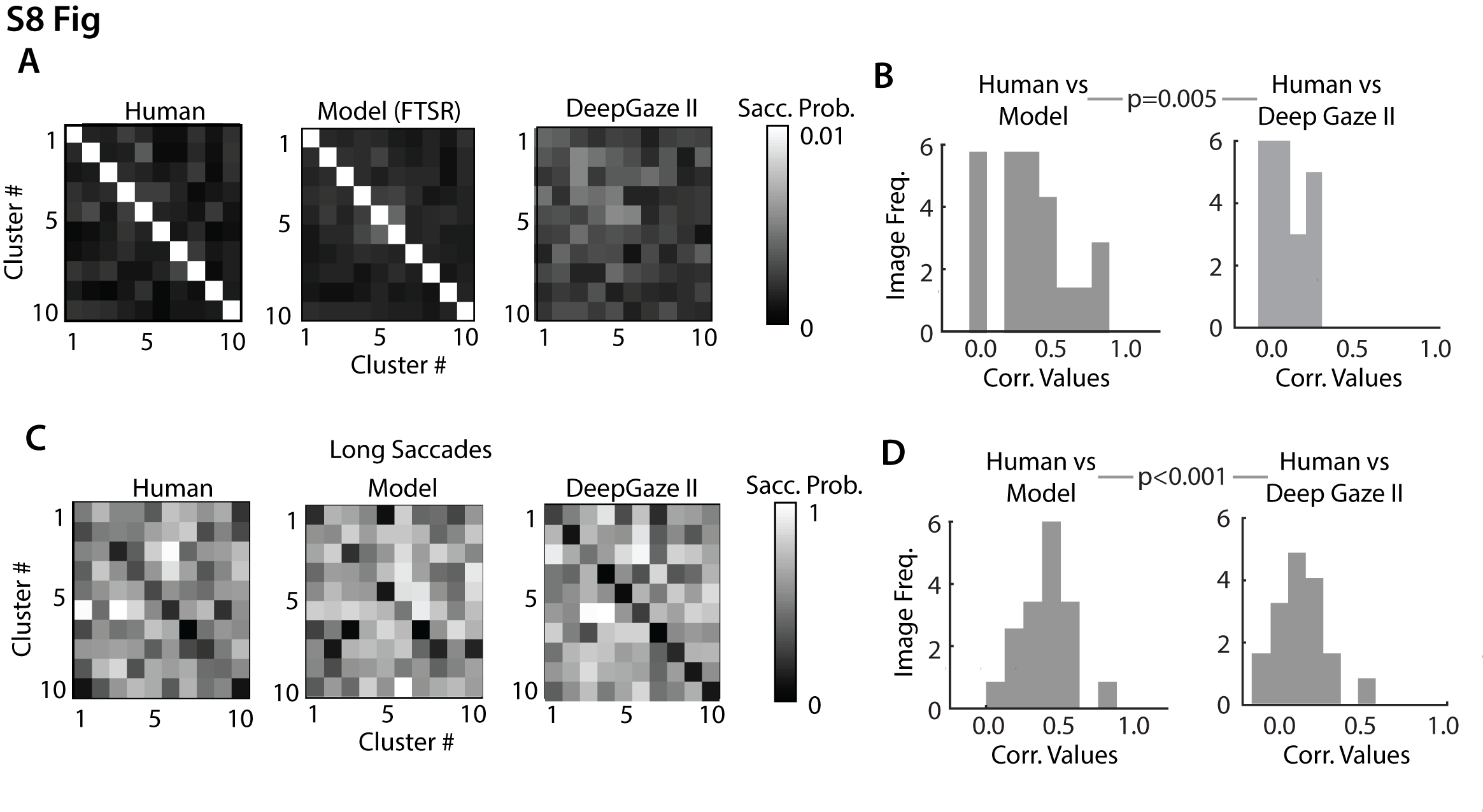

Supplement: S8 Fig — A-B. Same as in Fig 7C and 7D (main text), except with replacing DeepGaze’s saliency algorithm with the frequency-tuned salient region detection algorithm. C-D. Same as in Fig 7C and 7D (main text) except including only saccades whose amplitude was at least as large (or greater) than the 10th percentile of saccade amplitudes generated by the DeepGaze model (Fig 7B, main text, dashed vertical line). For C, the saccade probability matrix was normalized by its range for visualization purposes only. Other conventions are the same as in Fig 7C and 7D (main text). (TIF) [file pcbi.1009322.s008.tif]
