# Supplementary material for: Neurally-constrained modeling of human gaze strategies in a change blindness task
Source: PLoS Comput Biol. 2021 Aug 24;17(8):e1009322. doi: 10.1371/journal.pcbi.1009322 (PMC8478260; doi:10.1371/journal.pcbi.1009322)
Supplement: S1 Table — (DOCX) [file pcbi.1009322.s009.docx]

Supporting Information for

## Neurally-constrained modeling of human gaze strategies in a change blindness task­

### Supplementary Table

### S1 Table. List of images employed in the change blindness task

| **ID** | **Image** | **Type of Change** |
| --- | --- | --- |
| 01 | Engine Room | Appearance/ Disappearance |
| 02 | Fruitseller | Change in Size |
| 03 | Garden Ed | Appearance/ Disappearance |
| 04 | Holyrood | Appearance/ Disappearance |
| 05 | Illiterati | Appearance/ Disappearance |
| 06 | Inveraray | Change in Shape |
| 07 | Madrid | Appearance/ Disappearance |
| 08 | Market Venice | Appearance/ Disappearance |
| 09 | Meadows | Appearance/ Disappearance |
| 10 | Park Sale | Appearance/ Disappearance |
| 11 | Outdoor Party | Appearance/ Disappearance |
| 12 | Pavilion Café | Appearance/ Disappearance |
| 13 | Plaza Mayor | Change in Shape |
| 14 | Retiro Park | Appearance/ Disappearance |
| 15 | Ride Zone | Change in Color |
| 16 | Souvenir Shop | Change in Color |
| 17 | Jewellery | Change in Color |
| 18 | Visitors | Change in Color |
| 19 | Vintage Sale | Appearance/ Disappearance |
| 20 | Vatican City | Appearance/ Disappearance |
