# Supplementary material for: Neurally-constrained modeling of human gaze strategies in a change blindness task
Source: PLoS Comput Biol. 2021 Aug 24;17(8):e1009322. doi: 10.1371/journal.pcbi.1009322 (PMC8478260; doi:10.1371/journal.pcbi.1009322)
Supplement: Attachment — Submitted filename: 2021-04 Paper Review.docx [file pcbi.1009322.s010.docx]

In this paper, the authors find a large difference in performance on change detection, and clearly establish its correlation with easily measured behavioral metrics (mean fixation duration and saccade amplitude). Overall, the paper is very well written. Though other reviewers likely have comments on the experimental results, I find them really interesting and compelling. The paper could stand on its own with only the experimental results. The addition of the computational model is what makes it relevant for Plos Comp Bio. While the computational model has a lot of potential, its presentation needs considerable work. My major comments are centered on the modeling.

Major Comments

- In assessing the model, there is a tendency to conflate saccade statistics and change detection. Those need to be separated better, as they need not correlate. For example, how do the saccade metrics for the model in the first image compare to those of the second image? Or, what alternative models are there for change detection and how does this model compare to them? (Deepgaze is presented for saccade statistics.)
- The comparison with Deepgaze in the main text is misleading.
  - Going into the methods, it appears the model is really Deepgaze + novel additions, the most important of those being a stopping rule for saccade generation in the second image. It is thus not a clean comparison to say the model is better than Deepgaze, as the model is really Deepgaze with additional features.
  - Given that the most clear difference between the new model and standard Deepgaze is the stopping rule, it is confounding to include the second image in the saccade comparison. I get that they only run Deepgaze for the median number of timesteps the model with the stopping rule utilizes. That confounds though with saccade order.
  - Saccade order brings us to the next point. In figure 6E, the lack of a null diagonal for Deepgaze makes me think that Deepgaze is saccading to the same location. How does that make sense? That alone could confound the timestep restriction.
  - For a true comparison with Deepgaze, a few things need to happen.
    1. It needs to be made abundantly clear in the main text that the new model includes Deepgaze for saliency computation. That can’t be buried in the methods, as it feels like something is being hidden.
    2. The comparison needs to be bifurcated between the second image and the first image. That controls for the confound of the stopping rule.
    3. There needs to be a more “good-faith,” effort at translating the saliency map Deepgaze generates into saccades. At the very least, the Deepgaze model needs an “inhibition of return,” (similar to Itti, Koch, Vision Research, 2000).
- The framing of the model needs to change.
  - At this moment, I don’t see a gain in mechanistic insight. For example, if altering the foveal magnification causes greater amplitudes or changes in mean duration, that would provide mechanistic insight. As it stands, changing the mean duration parameter in the model results in the same observed performance differences as humans. While that tells me the model is doing a good job recapitulating observed experiments, it provides no real insight as to the mechanisms underlying human performance.
  - Next, the null models (lesions of the Bayesian SPRT rule starting on line 487) have no justification beyond that they are limits on the proposed model. There’s no discussion that the lesions to the model reflect plausible mechanisms. This is a necessary step to prove a functional stopping rule (such as the SPRT) is important, could an alternative stopping rule achieve the same results? Answering this question could provide mechanistic insight.
  - What I see right now is a validation of a novel model for predicting saccade patterns. That itself is important, and is worth leaning into. If that’s what the model is getting at, are there other datasets it can be compared against? This might be a big, time-consuming addition, but if the model is able to account for more experimental results than just those of the group, the impact of the model is greatly expanded.

Minor Comments

- Why use an SVM with a polynomial kernel? If the goal is feature importance and a nonlinear classifier, random forest (with Matlab’s Treebagger) with feature permutation would be more straightforward. It is less sensitive outliers and noninformative features. Furthermore, feature permutation as a measure of feature importance is a highly validated metric and relatively straightforward. I don’t anticipate the result would be different, but using that method could be easier for readers to understand and trust. It would probably only take an afternoon or so to implement in Treebagger, as you already have the processing pipeline for SVM in Matlab.
- Figure 1B, cleanup the location of the dashed lines. Looks messy.
- Show the null distribution (shuffled labels) in the figure 1C, not just the chance level.
- Figure 3A, could be useful to show a subtraction of the matrices. Not necessary, but would clarify the point.
- Figure 4C/D Is there a way to visually connect the colors? I shouldn’t have to look to the legend to understand the connection.
- In many figures (Figure 6 being the worst) the order of the panels is really confusing. They should be laid out sequentially.
- Line 330 and line 331, “In other words,” is used twice.
- Please provide more numbering of equations in the methods section. That will make it easier when the paper is published for people to use it.
