# Supplementary material for: Neurally-constrained modeling of human gaze strategies in a change blindness task
Source: PLoS Comput Biol. 2021 Aug 24;17(8):e1009322. doi: 10.1371/journal.pcbi.1009322 (PMC8478260; doi:10.1371/journal.pcbi.1009322)
Supplement: Attachment — Submitted filename: Rebuttal v4.docx [file pcbi.1009322.s011.docx]

**Responses to the reviewers**

We thank the reviewers for the insightful comments. Addressing these comments has significantly improved the quality of the revised manuscript.

**Reviewer #1**

Authors have done an interesting study on the pattern of saccades for detection of change in consecutive images and showed that there is a variability in the performance of subjects. Besides, they provided a model based on evidence accumulation that they claimed has better performance than Deep gaze neural networks. I believe that though the authors did a very good job in their analyses and modeling, but there is some gaps between behavioral model and study that should be addressed. my suggested extra analyses can be listed as below:

We thank the reviewer for the positive assessment.

We have made several major changes to the model and the analyses, based on the reviewer’s suggestions. These are summarized below, and expanded subsequently:

1) We have performed analyses that compare the distribution of fixation durations and frequency of fixation locations relative to the change location, for good and poor performers.

2) We have compared the time to first saccade to the change location and time to change detection across good and poor performers.

3) We have made major updates to the model in terms of two parameters: i) incorporated a decaying threshold and ii) incorporated prior ratios into the evidence accumulation. We performed extensive simulations of the model with these new features to showcase putative mechanisms for the effect of fixation durations on change detection performance.

4) We have tested for multimodality of the fixation duration and saccade amplitude distributions with Gaussian Mixture Models to identify differences in modes across good and poor performers.

5) We have re-evaluated our comparison of our model with the DeepGaze model by considering only long saccades generated by the DeepGaze model.

**1-** in behavioral analysis, though they clustered saccade related areas, but at each image, different clusters are associated with change location, so, we can't infer how much subjects fixated on areas near to change location. so please add an extra analysis that show good performers and poor performers how much fixated in areas near to change location; it can be shown by the total fixation duration and frequency at different locations based on the distance of fixation location relative to the center of change location.

We have now performed this analysis. We observed no systematic differences between good and poor performers in the distributions of either total fixation duration or frequency of fixations, relative to the change location. The results are presented in SI Fig. S4 of the revised manuscript.

**SI Figure S4**

We have revised the text, as follows:

“Fifth, we tested whether good and poor performers differed systematically in the spatial distributions of fixations relative to the change location, before change was detected. For this, we computed the frequency of fixations and the total fixation duration, based on the distance of fixation relative to the center of the change location (binned in concentric circular windows of increasing radii, in steps of 50 pixels, Methods). We observed no systematic differences in the distributions of either total fixation duration, or frequency of fixations, relative to the change location between good and poor performers (SI Fig. S4; p=0.99 for fixation duration, p=0.97 for fixation frequency, Kolmogorov-Smirnov test). In other words, the spatial distribution of fixations, relative to the change location, was similar between good and poor performers.”

The full set of distributions for each image and subject is available in the supplemental data file included in link provided in the “Data availability” section.

**2-** the time to first saccade to change location and also the time to fixate 3 sec on change area can be provided for each image and each subject. it can help to analyze the behavior relative to model; because if we see poor performers fixate on change location but don't detect the change, it means that fixation duration or drift rate were not enough for change detection or their threshold was high; but if they did not fixate on areas near change location it means that their weakness is on finding relevant area.

We have now performed this analysis also. Again, we observed no significant differences in the time from trial onset to first saccade to change location, or the time from trial onset to detect the change (3s fixation on change area), between good and poor performers. The results are presented in Figs. 2G-H of the revised manuscript. The full set of distributions for each image and subject is available in the supplemental data file included in link provided in the “Data availability” section.

Taken together with the previous analysis these results indicate that *poor performers fixated as often and as close to areas near the change, but simply failed to detect these changes successfully.*

**Figure 2**

We have revised the text, as follows:

“Finally, we tested whether good and poor performers differed in the time to first fixation on the region of change, or the time to detect changes (on successful trials). Again, we observed no significant differences in the distributions of either time to first fixation, or time to detect changes, between good and poor performers (Figs. 2G-H; p=0.08 for time to first fixation in change region, p=0.28 for time to detect change, signrank test). Taken together with the previous analysis, these results indicate that poor performers fixated as often and as close to areas near the change, but simply failed to detect these changes successfully.

Overall, these analyses indicate that relatively simple gaze metrics like fixation durations and saccade amplitudes predicted successful change detection. More complex metrics like scan paths, fixated image features or the spatial distribution of fixations, were not useful indicators of change detection success. In other words, “low-level” gaze metrics, rather than “high-level” scanning strategies, determined participants’ success with change detection.”

**3-** one hypothesis can be that the threshold is decaying with different rate in different subjects and so subjects with lower decaying fixate more on each location, so it will be great if you can add threshold decaying that can account for an important mechanism in the model for poor performers.

We thank the reviewer for these suggestions. We have now added multiple novel elements to the model:

i) Threshold decaying with different rates: We have incorporated a no-change threshold for breaking fixations in the model. Fixation intervals are no longer sampled from a Gaussian. Rather, if the accumulated evidence crosses a negative (no-change) threshold F_n_ (Table 1), the observer terminates the current fixation. We decayed the no-change threshold with different decay rates and studied its effect on model performance.

We show that a faster decay of the no-change bound, possibly reflecting a stronger “urgency” to break fixations, resulted in faster bound crossing and, therefore, shorter fixations, and directly impacts performance (Fig. 5D, upper inset). This provides a plausible mechanism for shorter fixation durations in poor performers.

ii) Prior odds ratio: We have incorporated prior ratios into the likelihood-ratio based decision rule in the model (equation 2, revised manuscript). The prior odds ratio (P(C:NC)) reflects an individual’s subjective belief in the prior probability of change to no change. With this modification, our formulation of an SPRT decision involves evaluating and integrating fully the logarithm of the Bayesian posterior odds ratio (product of the prior odds and likelihood ratio).

We demonstrate that a lower prior odds ratio (higher degree of belief in no change) biases the evidence accumulation process toward the negative threshold, and leads to shorter fixations, and impacts performance (Fig. 5D, lower inset). This provides a second, plausible mechanism for shorter fixation durations in poor performers.

In summary: we no longer sample fixation durations from a Gaussian but, per the reviewer’s suggestion, permit variations in fixation durations to emerge naturally from variations in the decaying threshold, or prior ratios.

The details are provided in the Results and Fig. 5 in the revised manuscript.

Another key change to the model involves studying the effect of the foveal magnification factor (FMF) on saccade amplitude variance (Fig. 5F in the revised manuscript). These revisions are described in more detail in Response #III-b to Reviewer #2.

**Figure 5**

We have revised our Results, when describing the model, as follows:

“Second, the observer integrates the “evidence” for change at location *A_i_*, by accumulating the logarithm of the likelihood ratio log(*L_i_(t)*), **along with the log of the prior odds ratio (*P_i_*),** as in the SPRT framework.

$E_{i}\left( t \right)=\left( 1-\gamma_{i}\left( t \right) \right)E_{i}\left( t-1 \right)+{log(L}_{i}\left( t \right))+\boldsymbol{log}\left( \boldsymbol{P}_{\boldsymbol{i}} \right)+W_{i}\left( t \right)$	… (2)

where *ɣ_i_* ∈[0,1] is a decay parameter for evidence accumulation at location *A_i_*, which simulates “leaky” evidence accumulation (15,29) with larger values of *ɣ_i_*, indicating greater “leak” in evidence accumulation**, *P_i_* is the prior odds ratio of change to no change (P(C)/P(N)) at each location**, *W_i_(t)* represents white noise*,* sampled from a uniform distribution (Table 1), to mimic noisy evidence accumulation (30). Here we assume that the prior ratio is constant across time and space, but nonetheless study the effect of varying prior ratios on model performance (next section).

Both of these features – leak and noise in evidence accumulation – are routinely incorporated in models of human decision-making (29), and are grounded in experimental observations in brain regions implicated in decision-making (15). Evidence accumulation occurs in the original, physical space of the image, and not in the CVR transformed space (Fig. 4B). **Note that this formulation of an SPRT decision involves evaluating and integrating fully the logarithm of the Bayesian posterior odds ratio (product of the prior odds and likelihood ratio, *P_i_ × L_i_(t)*).**

Evidence accumulation is performed for each region in the image; $E_{i}(t)$ for each region is calculated independently of the other regions. If the accumulated evidence $E_{i}(t)$ crosses a positive threshold, *F_c_* (Table 1), the observer stops scanning the image and region *A_i_*, at which the threshold *F_c_* was crossed, is declared the “change region”. **If, on the other hand, the accumulated evidence crosses a negative (no-change) threshold *F_n_* (Table 1),** the observer terminates the current fixation and determines the next region to fixate, *A_k_*, based on a softmax probability function:

$p_{k} ={e^{\frac{E_{k}}{T}}/\Sigma}_{i=1}^{N}e^{\frac{E_{i}}{T}}$	… (3)

where *E_i_* is the evidence value for region *i*, *N* is the number of regions in the image, and T is a temperature parameter which controls the stochasticity of the saccade (decision) policy (Methods; see also next section). For selecting the next point of gaze fixation, we also matched directional saccadic biases typically observed in human data (31) (Fig. 4B, described in Methods section on “Comparison of model performance with human data**”).**

**In some simulations we also decayed the no-change threshold (*F_n_*) with different decay rates (ζ; Table 1) and studied its effect on model performance. Because we observed virtually no false-alarms (signaling a no-change location as change) in our experimental data (0.06% of all trials; Methods) we did not model decay in the change threshold (*F_c_*), which would have yielded significantly more false alarms.”**

And when describing the effects of parameter variations in the Results, we have revised the text, as follows:

“Fourth, we tested the effect of varying mean fixation duration (μ_FD_) – a key parameter identified in this study as being predictive of success with change detection. The mean fixation duration is not a parameter of the model. We, therefore, varied the mean fixation duration, indirectly, by varying the prior odds ratio (*P*) and the decay rate (ζ) of the no-change threshold (*F_n_*). A lower prior odds ratio of change to no-change biases evidence accumulation toward the no-change threshold, leading to shorter fixations (and vice versa; Fig 5D, lower inset).

On the other hand, a higher decay rate of the no-change threshold leads to a greater probability of bound crossing of the evidence in the negative direction, again leading to shorter fixations (and vice versa; Fig. 5D; upper inset). In either case, we found that decreasing (increasing) the mean fixation duration produced systematic deterioration (improvement) in the performance of the model (Fig. 5D). These results recapitulate trends in the human data, indicating that increased fixation duration may be a key gaze metric indicating change detection success.

Fifth, we tested the effect of varying the saccade amplitude variance ($\sigma_{SD}^{2}$) – the other key parameter we had identified as being predictive of change detection success. Again, because the variance of the saccade amplitude is not a parameter of the model, we varied this, indirectly, by varying the temperature (*T*) parameter in the softmax function: a higher temperature value leads to random sampling from many regions of the image, thereby increasing $\sigma_{SD}^{2}$ whereas a low temperature value leads to more deterministic sampling, thereby reducing $\sigma_{SD}^{2}$ (Fig. 5E, inset). With increasing saccade variance, performance dropped steeply (Fig. 5E). Finally, we also explored the effect of varying the foveal magnification factor (FMF) across a two-fold range. Saccade amplitude variance decreased robustly as the FMF increases (Fig. 5F, inset) (see Discussion). As with the previous simulation, we observed a systematic decrease in performance with increasing saccade amplitude variance, again, recapitulating trends in the human data.”

We have added more content in the revised Discussion, further elaborating on these results:

“Finally, the model provided mechanistic insights about key trends observed in our own experiments, specifically, a critical dependence of success rates on mean fixation durations and the variance of saccade amplitudes (Figs. 5D-F). Fixation durations in the model varied systematically either with altering the prior odds ratio or the decay rate of the no-change bound. Note that the prior odds ratio correspond to an individual’s prior belief in the prior probability of change to no change. The lower this ratio, the higher the degree of belief in no change, and the sooner the individual seeks to break each fixation. In our model, this was achieved by having the prior ratio biased evidence accumulation toward the no-change (negative) bound. Similarly, faster decay of the no-change bound, possibly reflecting a stronger “urgency” to break fixations, resulted in faster bound crossing and, therefore, shorter fixations.

Regardless of the mechanism, shorter fixation durations resulted in impaired change detection performance (Fig. 5E), providing a putative mechanistic link between fixation durations and change detection success in the experimental data. In addition, saccade amplitude variance modulated systematically with changes in the foveal magnification factor (FMF). We propose that this happens because with higher foveal magnification, the model is able to better perceive features in regions proximal to the fixation location, and saccade to them, thereby resulting in overall shorter saccades, and lower variance. Moreover, the higher foveal magnification, enables analyzing the region of change with higher resolution, thereby leading to better change detection performance. As a consequence performance degraded systematically with increased saccade amplitude variance (Fig. 5F), the common underlying cause for each being the change in the foveal magnification factor. This provides a plausible mechanism for higher variance of saccade amplitudes in “poor” performers.”

**4-** in feature list, saccade amplitude variance should be divided to its components; in other words, because saccade amplitude distribution probably is bimodal and short and long saccades have different means and also different probability, using variance cause to miss important information on the strategy of subjects, so please fit distribution of saccade amplitude by gaussian mixture model and investigate the mean of each cluster and their frequency. please check bimodality for fixation duration too, if it is bimodal the same issue should be checked for fixations before large saccade and small saccades.

We performed this analysis also. The saccade amplitude distribution was plotted for individual subjects and found to be well-fit with log-normal distributions. We, therefore, fitted the logarithm of the saccade amplitudes with a Gaussian mixture model, and performed two analyses.

First, we performed a two-way anova with good and poor performers as categorical factors, and the number of mixture components as continuous factors. Yet, we did not find any significant evidence for multimodality in these distributions: the Bayesian Information Criteria (BIC) for a GMM with 1-5 components were not significantly different from each other (F(1, 101)=0.01, p=0.91 for a main effect of number of components).

We observed confirmatory evidence with Hartigan’s “dip” test of unimodality: The test failed to show evidence for bimodality at the p=0.05 level in all 9/9 good performers (median p=0.99) and in 10/12 poor performers (median p=0.99). These results indicate that statistical tests overwhelmingly favored a unimodal distribution of saccade amplitude across both classes of performers.

**SI Figure S2B**

The plot above shows the saccade amplitude distributions for representative individual subjects (pooled across all images), with p-values from Hartigan’s dip test for unimodality. The full set of distribution for each image and subject is available in a supplemental data file included in the “Data availability” link.

A similar analysis for fixation durations revealed no systematic evidence for bimodality: Again, BICs for a GMM with 1-5 components were not significantly different from each other (F(1, 101) = 0.01, p=0.94 for a main effect of number of components). Similarly, Hartigan’s test failed to show evidence for bimodality with p>0.05, in 8/9 good performers (median p=0.74), and in 8/12 poor performers (median p=0.31). Consequently, we did not pursue further analysis involving mixture modeling of the fixation durations.

The results are shown in SI Figure S2B. We have revised the text, as follows:

“We considered the possibility that the differences in fixation duration and saccade amplitude variance between good and poor performers could arise from differences in multiple, distinct modes of these, respective distributions. Nonetheless, statistical tests provided no significant evidence for multimodality in either fixation duration or saccade amplitude distributions for either class of performers (Hartigan’s dip test for unimodality; fixation duration: p>0.05, in 8/9 good performers with median p=0.74, and in 8/12 poor performers with median p=0.31; saccade amplitudes: p>0.05, in 9/9 good performers with median p=0.99 and in 10/12 poor performers with median p=0.99).”

**5-** study has two parts, first part studied individual differences that is very important and can extend this study for many applications such as clinical applications, but in modeling part analyses are mostly in images' difficulty (performance on each image). so the coherence of paper has been reduced by missing the link between model and individual differences; I understand that data is small and fitting on each subject has some difficulties, but using hierarchical bayesian modeling you can use data from all subjects and have two clusters of poor and good performers on that, and provide the value of each parameter for each subject, it will help to analyze what aspects of model caused to good or poor performance in different subjects. this will help to make paper more coherent and will help others to find important applications for your model to study individual differences.

We appreciate the reviewer’s excellent suggestion regarding estimating model parameters by fitting individual subjects’ eye movement data with maximum likelihood estimation, or fitting groups of individuals (good/poor) with hierarchical Bayesian modeling. Yet, key challenges with our model preclude such an analysis.

First, our model comprises over a dozen parameters (Table 1), but generates only two gaze parameter distributions: fixation duration and saccade amplitudes. In its current form, the model does not account for other gaze parameters like saccade speed or pupil diameter. It was challenging to fit this high-dimensional model with only two observed parameters. Second, the model is not identifiable: Multiple parameters within the model produce the same effects on gaze parameters. For example, changing either the prior ratio or the decay rate of the no change (negative) threshold (or both) systematically changes fixation durations (Fig. 5D, insets). Similarly, changing either the temperature parameter (T) of the softmax function (Fig. 5E) or the foveal magnification factor (Fig. 5F) (or both) systematically changes the saccade amplitude variance. As a consequence, ML estimation of model parameters yields multiple, redundant parameter configurations, all corresponding to the same gaze parameter distributions.

We understand the reviewer’s viewpoint in terms of fitting the model to data for finding applications of the model. We have amended the Discussion to reflect this viewpoint.

“Nonetheless, our SPRT model can be improved in a few ways. First, saliency maps in our model were typically computed with low-level features (e.g. Fig. 5; the frequency tuned salient region method). Incorporating more advanced saliency computations (e.g. semantic saliency) (51) into the saliency map could render the model more biologically realistic. Second, although our neurally-constrained model provides several biologically plausible mechanisms for explaining our experimental observations, it does not identify which of these mechanisms is actually at play in human subjects. To achieve this objective, model parameters may be fit with maximum likelihood estimation (52) or Bayesian methods for sparse data (e.g. hierarchical Bayesian modelling) (53). Yet, in its current form, such fitting is rendered challenging because the model is not identifiable: multiple parameters in the model (e.g. prior ratios or decay of the no-change threshold) produce similar effects on specific gaze parameters (e.g. fixation durations, Fig. 5D). Future extensions to the model, for example, by measuring and modeling more gaze metrics for constraining the model, may help overcome this challenge. Such model-fitting will find key applications for identifying latent factors contributing to inter-individual differences in change detection performance.”

**6-** to be more fair on deep gaze, it is good to compare it based on just large saccades, because it seems that deep gaze is good in predicting large saccades and not small saccades.

We have now performed this analysis. We show a comparison between gaze patterns for human observers, our model, and Deep Gaze using only large saccades (SI Fig. S8C-D). Specifically, we limited the model comparison to the top 10th percentile of saccades produced by the DeepGaze model (Fig. 7A, dashed vertical line).

**Figure 7**

**SI Figure S8**

We have revised the text, as follows:

“The chief reason for these differences was readily apparent upon examining the saccade amplitude distributions across the human data, our SPRT model and DeepGaze: whereas the human and model distributions contained many short saccades, the DeepGaze distribution contained primarily long saccades (SI Figs. S8 C-D). Consequently, we repeated the comparison of saccade probabilities limiting ourselves to the range of saccade amplitudes in the DeepGaze model. Again, we found that our model’s saccade probabilities were better correlated with human saccade probabilities (SI Figs. S8 C-D) (human-SPRT model: median r=0.29, human-DeepGaze II: median r=0.10; p<0.001). We propose that these differences occurred because DeepGaze saccades are generated based on relative saliencies of different regions across the image, whereas saliency computation, *per se,* may be insufficient to model human saccade strategies in change blindness tasks or, in general, in change detection tasks.”

**Minor points:**

in some of images your change is related to color change, but you have just mentioned normal vision, please add that they have not any color blindness too.

We have now amended this (line 847, redlined version).

in fig. S1, you said that circle highlighted... while in figure you have square for that.

Thank you, this has been amended. Per journal policies (which we were informed of during the revision) Fig. S1 been moved to the Data Availability link.

in the model, inhibition of return has not been included, it seems that it may help to enhance the model; as a suggestion if you'd like you can test it too.

Although inhibition of return has not been explicitly included, such a feature emerges naturally from the evidence accumulation rule. We have now clarified this in the revised Results:

“Note that although we have not explicitly modeled inhibition-of-return (IOR), this feature emerges naturally from the evidence accumulation rule in the model. Following each fixation, the accumulated evidence for no-change decays gradually (equation 2), thereby reducing the probability that subsequent fixations occur, immediately, at the erstwhile fixated location. This feature encourages the model to explore the image more thoroughly.”

**Reviewer #2**

In this paper, the authors find a large difference in performance on change detection, and clearly establish its correlation with easily measured behavioral metrics (mean fixation duration and saccade amplitude). Overall, the paper is very well written. Though other reviewers likely have comments on the experimental results, I find them really interesting and compelling. The paper could stand on its own with only the experimental results. The addition of the computational model is what makes it relevant for Plos Comp Bio.

We thank the reviewer for the positive assessment.

While the computational model has a lot of potential, its presentation needs considerable work. My major comments are centered on the modeling.

We list below key changes to the manuscript based on the reviewer’s comments, and provide details in subsequent responses:

1) We have improved the presentation of the model so that we clarify the relationship between saccade statistics and change detection in our data and model.

2) We have now performed a thorough and careful comparison with DeepGaze with multiple control analyses, including the addition of an inhibition-of-return (IOR) component to DeepGaze. We have also revised the text to present the comparison in a more balanced manner.

3) To obtain mechanistic insights, we study the effect of varying the foveal magnification factor (FMF) on model parameters. In addition, we have significantly updated the model in terms of two additional parameters: i) incorporated a decaying threshold and ii) incorporated prior ratios into the evidence accumulation. We performed extensive simulations of the model with these new features to obtain additional mechanistic insights to explain the effect of fixation durations on change detection performance.

5) We have now removed one of the null (control) models and now report comparisons with 3 alternative models with different search strategies or stopping rules, and have discussed plausible mechanisms underlying these control models.

6) We now report feature selection with the bag of decision trees (Treebagger) algorithm.

**Major Comments**

**I)** In assessing the model, there is a tendency to conflate saccade statistics and change detection. Those need to be separated better, as they need not correlate. For example, how do the saccade metrics for the model in the first image compare to those of the second image?

In general, saccade statistics and change detection need not be correlated. Yet, these are correlated, by design, in our task and study. The change blindness task requires subjects to make saccades with the specific goal of detecting changes by comparing the two images within a limited trial duration (60 s). Our model seeks to simulate these human gaze strategies for solving change blindness tasks. Consequently, saccades -- both in the human subjects and the model -- are made so as to localize changes (differences) between the two images.

In the model, the SPRT rule computes evidence for change in each region with information *from both images* and saccades are made to the location with the highest evidence for change, across the two images. As a result, change detection and saccade generation are inextricably intertwined -- they are part of the same framework in our study. Our experiments seek to identify gaze (saccade and fixation) metrics that can explain variations in change detection performance, and the model seeks to mimic gaze statistics directly relevant to the goal of change detection.

In the “flicker” paradigm employed in this study, as well as in previous change blindness studies (Simons and Rensink, 2005; Rensink, O’Regan and Clark, 1997), the first and second images alternate rapidly and repeatedly at a 4 Hz rate (Fig. 1A). These alternations (odd image/A + blank + even image/A’ + blank) occur every second, for 60 s (trial duration) or until the subject detects the change. The pair of images flicker rapidly and alternate many times on each trial. Because of the rapidity of the stimulus alternation, humans are unlikely to be able to plan and execute saccades over each image, independently.

Our model of this change blindness task *is not designed to generate saccades based on viewing any one of the two images alone*. The likelihood ratio computation (equation 1) depends critically on viewing each alternation, in turn: odd image (A) followed by the even image (A’), or vice versa.

Because this is also relevant to a later comment, we have clarified this point in considerably more detail in the response to #II-b, below.

Or, what alternative models are there for change detection and how does this model compare to them? (Deepgaze is presented for saccade statistics.)

There are several algorithms in literature that address the broader problem of “change point” detection (e.g. Adams and MacKay, 2007; Matteson and James, 2014; van den Burg et al, 2020). None of these algorithms are neurally-constrained (e.g. foveal magnification, Poisson statistics), and none models gaze information or saccades. To our knowledge, ours is the only neurally-constrained model for gaze strategies in change blindness tasks, and developing and validating such a model is a central goal of our paper.

We have clarified this element of novelty of our approach – developing a neurally constrained model of gaze for change detection – in the revised Discussion.

“On the one hand, our study follows a rich literature on human gaze models, that fall, loosely, into two classes. The first class of “static” models use information in visual saliency maps (23,37,39) to predict gaze fixations. These saliency models, however, do not capture dynamic parameters of human eye fixations, which are important for understanding strategies underlying visual exploration in search tasks, like change blindness tasks. The second class of “dynamic” models seek to predict the temporal sequence of gaze shifts (40–43). Nevertheless, these approaches were developed for free-viewing paradigms, and comparatively few studies have focused on gaze sequence prediction during search tasks (44,45). On the other hand, several previous studies have developed algorithms to address the broader problem of “change point” detection (46–48). Yet, none of these algorithms are neurally-constrained (e.g. foveal magnification, Poisson statistics), and none models gaze information or saccades. To the best of our knowledge, ours is the first neurally-constrained model for gaze strategies in change blindness tasks, and developing and validating such a model is a central goal of this study.

.”

To address the reviewer’s suggestion regarding alternative models, we compare the SPRT model with alternative models with different search strategies or different stopping rules. We show that these alternative models fare relatively poorly as compared to the SPRT rule. The details are provided in the response to comment #III-c.

**II)** The comparison with Deepgaze in the main text is misleading.

We apologize for being insufficiently clear with describing our comparison. We have rectified this shortcoming by improving the description in the revision, and have also

performed the suggested control analyses.

**II-a)** Going into the methods, it appears the model is really Deepgaze + novel additions, the most important of those being a stopping rule for saccade generation in the second image. It is thus not a clean comparison to say the model is better than Deepgaze, as the model is really Deepgaze with additional features.

For all simulations shown in Figure 5 we employed the frequency-tuned salient region detection (FTSR) algorithm (Achanta et al, CVPR, 2009). For performing the comparison with DeepGaze alone (previous Figs. 6E-F, revised Figs. 7C-D), we had used DeepGaze’s own saliency map with our model, so that a direct comparison could be made between saccades generated by our model and those generated by DeepGaze. These aspects are now presented, upfront in the respective sections of the Results (lines 485-486, and lines 576-580, redlined manuscript).

To demonstrate that our model does not depend on the DeepGaze saliency algorithm for its success, we repeated the analysis by replacing DeepGaze’s saliency algorithm with the FTSR algorithm that we used in the other simulations. With this replacement, show that our model is superior in terms of its match to human gaze data (SI Fig. S8A-B in the revised manuscript).

We have revised the text as follows:

“Confirming these trends, we observed significantly higher correlations between the human saccade probabilities and our model’s saccade probabilities (Fig. 7D, left) as compared to those with DeepGaze’s saccade probabilities (Fig. 7D, right) (human-SPRT model: median r=0.51, human-DeepGaze II: median r=0.14; p<0.01 for significant difference in correlation values across n=20 images, signed rank test). **These results were robust to the underlying saliency map in our SPRT model: replacing DeepGaze’s saliency map with the frequency-tuned salient region detection method yielded nearly identical results (SI Fig. S8 A-B).**”

**SI Figure S8 A-B**

We have also made key changes to the DeepGaze simulations to permit a fairer comparison. These changes, and the modifications to the text incorporating these revisions, are described in the response to comment #II-d.

**II-b)** Given that the most clear difference between the new model and standard Deepgaze is the stopping rule, it is confounding to include the second image in the saccade comparison. I get that they only run Deepgaze for the median number of timesteps the model with the stopping rule utilizes. That confounds though with saccade order.

Unlike DeepGaze, our model is not designed to generate saccades *by viewing only one of the two images alone* because it relies on a likelihood ratio-based SPRT rule (equation 1) for generating saccades. For computing the likelihood ratio for change versus no change, the model must look for differences in each region *across the two images* (either A followed by A’ or vice versa, Fig. 1A) and generate the saccade based on the likelihood ratio.

We have clarified this in the revised Results:

“For ease of understanding we summarize key steps in our model’s saccade generation pipeline (Figs. 4A-B), first; a detailed description is provided thereafter.

In the model, distinct neural populations, with (noisy) Poisson firing statistics, encode the saliency of the foveally-magnified image at each region. During fixation, following each alternation (Fig. 1A, either A followed by A’, or vice versa) the model computes a posterior odds ratio for change versus no change at each region and at each instant of time (equation 1), and accumulates this ratio as “evidence” (equation 2, Results). If the accumulated evidence exceeds a predetermined (positive) threshold for change detection at the location of fixation, the model is deemed to have detected the change. If, the accumulated evidence dips below a predetermined (negative) threshold for “no change” at the fixated location, the observer terminates the current fixation. The next fixation location is chosen based on a stochastic (softmax) decision rule (equation 3), with the probability of saccade to a region being proportional to the accumulated evidence at that region. Note that both images -- odd and even -- must be included in these computations to generate each saccade. The model continues scanning over the images in the sequence until either the change is detected or until the trial duration has elapsed (as in our experiment), whichever occurs earlier.”

In contrast, as the reviewer correctly notes, DeepGaze generates saccades independently on the two images. Our primary goal with the comparison with DeepGaze is to demonstrate that even state-of-the-art free-viewing saliency prediction algorithms are not successful with predicting gaze in a change detection/change blindness task setting. Saccades made with the goal of detecting changes are likely to be different from saccades made in free-viewing conditions.

We have also clarified our objective and sought to make a more balanced comparison with DeepGaze in the revised Discussion:

“Specifically, our model outperformed a state-of-the-art deep neural network (DeepGaze II), in terms of predicting saccade patterns in this change blindness task. Yet, a key difference must be noted when comparing our model with DeepGaze. Our model relies on a decision rule based on posterior odds for generating saccades: For this, it must compare evidence for change versus no change across the two images. In our simulations, in contrast, the DeepGaze model generates saccades independently on the two images, without comparing them. Based on these simulations, we found that our model’s gaze patterns provided a closer match to human data compared to gaze patterns from DeepGaze (Fig. 7C-D, SI Fig. S8). Because DeepGaze is a model tailored for predicting free-viewing saccades, this comparison serves only to show that even a state-of-the-art free-viewing saliency prediction algorithm is not sufficient to accurately predict gaze patterns in the context of a change detection (or change blindness) task. In other words, saccades made with the goal of detecting changes are likely to be different from saccades made in free-viewing conditions.”

Finally, we have clarified in the both the Abstract and the Introduction that the better performance of our model as compared to DeepGaze, is specific to the context of change blindness tasks.

Revised Abstract:

“Moreover, the model outperformed a state-of-the-art deep neural network (DeepGaze II) with predicting human gaze patterns **in this change blindness task.**”

Revised Introduction (last para):

“Finally, the model outperformed a state-of-the-art deep neural network (DeepGaze II (17)) in predicting probabilistic patterns in human saccades **in this change blindness task.**”

**II-c)** Saccade order brings us to the next point. In figure 6E, the lack of a null diagonal for Deepgaze makes me think that Deepgaze is saccading to the same location. How does that make sense? That alone could confound the timestep restriction.

We seek to clarify this point: DeepGaze actually has a *lower* diagonal value than our model and the human data. We reproduce the figure from our original submission below.

**Figure 6E (original submission)**

We plot the distribution of saccade amplitudes in each case, which clarifies this trend. Our SPRT model and humans make many short saccades, which yields high values on the diagonal. In contrast, DeepGaze primarily makes long saccades, which yields low values on the diagonal.

**Figure 7**

A plot with overlaid saccades (arrows) from the DeepGaze model on a representative image, is shown below, for reference. Rather than saccading to the same location, DeepGaze makes many long saccades.

Considering these differences, we have performed a control analysis removing the shortest saccades, and considering only relatively long saccades (largest 90th percentile of saccade amplitudes) generated by DeepGaze (dashed vertical line in revised Fig. 7A). The results are shown in SI Fig. S8C-D (revised manuscript). The revisions to the text are presented in detail in the next response (#II-d).

**II-d)** For a true comparison with Deepgaze, a few things need to happen.

1. It needs to be made abundantly clear in the main text that the new model includes Deepgaze for saliency computation. That can’t be buried in the methods, as it feels like something is being hidden.

As mentioned in response #II-a, we have mentioned this upfront in the Results (lines 576-580, redlined manuscript). We have also repeated the analysis by replacing the DeepGaze algorithm with the frequency-tuned salient region detection algorithm (SI Fig. S8 C-D).

2. The comparison needs to be bifurcated between the second image and the first image. That controls for the confound of the stopping rule.

As mentioned in response #II-b, our model is not designed to make saccades based on viewing one image alone, precluding such a comparison.

3. There needs to be a more “good-faith,” effort at translating the saliency map Deepgaze generates into saccades. At the very least, the Deepgaze model needs an “inhibition of return,” (similar to Itti, Koch, Vision Research, 2000).

Per the reviewer’s suggestions, we have made several changes to enable a fairer comparison with DeepGaze.

i) we have incorporated inhibition-of-return (IOR) in the DeepGaze model (following Itti & Koch, Vision Research, 2000). All of the comparisons in the revised manuscript are now made with the DeepGaze+IOR model (revised Methods, lines 1280-1287, redlined manuscript).

ii) rather than matching the median number of timesteps in each trial, we have “identically” matched the timing and duration of fixation interval distributions (timesteps for each fixation) between our SPRT model and DeepGaze (revised Methods, lines 1288-1292, redlined manuscript).

iii) we have performed a control analysis removing the shortest saccades, and considering only relatively long saccades (largest 90th percentile of saccade amplitudes) generated by DeepGaze (SI Figs. S8 C-D).

We have revised the text with these new changes, as follows:

Revisions to Methods:

“To enable a fair comparison with DeepGaze we incorporated the following additional features in the DeepGaze model simulations. First, inhibition-of-return (IOR) is an emergent feature of our model (see Results). We, therefore, incorporated IOR in the DeepGaze model as well (32). IOR was implemented as a Gaussian patch (G) centered on the current fixation (x, y) with a standard deviation (σ) of 20 pixels. The amplitude of G was scaled up by a time dependent factor (tanh(0.05 t)), so that the impact of IOR increased progressively over the course of the trial. IOR values were accumulated in a spatial map with a discount factor of 0.25 across successive timesteps (IOR(x, y, t) = (0.25 * IOR(x, y, t - 1)) + G(x, y; σ)). IOR values were clipped between 0 and 1, and the IOR map was subtracted from the foveally-magnified saliency map before computing the next location of fixation.

Second, because the DeepGaze model was not accumulating evidence for change, there was no clear termination criterion. Therefore, we identically matched the timing and distribution of fixation interval durations (timesteps for each fixation) with our SPRT model. This was accomplished by initiating and terminating each fixation in the DeepGaze model at the exact same times when these were initiated or terminated in the SPRT model, respectively.

Third, to ensure that both the model and DeepGaze produced saccades with the same level of stochasticity we identically matched the temperature parameter in the softmax function (equation 3) for deciding the next saccade location.

Lastly, we also performed comparisons with the human data by limiting the saccade amplitude range for comparison. The SPRT model (and humans) make many short saccades, whereas DeepGaze primarily makes long saccades (Fig. 7B). Therefore, we performed a control analysis, comparing the human data, SPRT model and DeepGaze considering only saccades with amplitudes greater than the 10th percentile of those generated by the DeepGaze model (SI Fig. 8C-D).”

Revisions to Results:

“Finally, we tested whether model gaze patterns would match human gaze patterns beyond that achieved by state-of-the-art fixation prediction with a deep neural network: DeepGaze II (17). First, we quantified human gaze patterns by computing the probability of saccades pair-wise among the top 10 clusters with the largest number of fixations (e.g. Fig. 7B) for each image. We then compared these human saccade probability matrices (Fig.7C, left) with those derived from simulating the model (Fig. 7C, middle) as well as with those generated by the DeepGaze network (Fig. 7C, right). For the latter, saccades were simulated using the same softmax rule as employed in our model (equation 3) along with inhibition-of-return (32) (Methods); in addition, for each image, we identically matched the distribution of fixation durations between DeepGaze and our model (Methods).

**Figure 7**

The model’s saccade probability matrix (Fig. 7C, middle) closely resembled the human saccade probability matrix (Fig. 7C, left), indicating that model was able to mimic human saccades patterns closely. On the other hand, the DeepGaze saccade matrix (Fig. 7C, right) deviated significantly from the human saccade probability matrix (Fig. 6E, upper). Confirming these trends, we observed significantly higher correlations between the human saccade probabilities and our model’s saccade probabilities (Fig. 7D, left) as compared to those with DeepGaze’s saccade probabilities (Fig. 7D, right) (human-SPRT model: median r=0.51, human-DeepGaze II: median r=0.14; p<0.01 for significant difference in correlation values across n=20 images, signed rank test). These results were robust to the underlying saliency map in our SPRT model: replacing DeepGaze’s saliency map with the frequency-tuned salient region detection method yielded nearly identical results (SI Fig. S8 A-B).

The chief reason for these differences was readily apparent upon examining the saccade amplitude distributions across the human data, our SPRT model and DeepGaze: whereas the human and model distributions contained many short saccades, the DeepGaze distribution contained primarily long saccades (SI Figs. S8 C-D). Consequently, we repeated the comparison of saccade probabilities limiting ourselves to the range of saccade amplitudes in the DeepGaze model. Again, we found that our model’s saccade probabilities were better correlated with human saccade probabilities (SI Figs. S8 C-D) (human-SPRT model: median r=0.29, human-DeepGaze II: median r=0.10; p<0.001). We propose that these differences occurred because DeepGaze saccades are generated based on relative saliencies of different regions across the image, whereas saliency computation, *per se,* may be insufficient to model human saccade strategies in change blindness tasks or, in general, in change detection tasks.”

**SI Figure S8 C-D**

**III)** The framing of the model needs to change.

**III-a)** At this moment, I don’t see a gain in mechanistic insight. For example, if altering the foveal magnification causes greater amplitudes or changes in mean duration, that would provide mechanistic insight. As it stands, changing the mean duration parameter in the model results in the same observed performance differences as humans. While that tells me the model is doing a good job recapitulating observed experiments, it provides no real insight as to the mechanisms underlying human performance.

Thank you for these suggestions.

We have now made 3 key changes to the model (also incorporating suggestions from Reviewer #1), each of which provides essential insights into mechanisms underlying human performance. We summarize these here, and elaborate on the textual revisions subsequently.

i) Foveal magnification factor: First, as per the reviewer’s suggestion, we varied the foveal magnification factor (FMF). Because our model requires expensive saliency map computations on the foveally-magnified image representation at multiple locations fixated on each image, we explored two values of the FMF (0.025 and 0.05).

We show that saccade amplitude variance decreases robustly as the FMF increases (Fig. 5F, inset). This happens likely because with higher foveal magnification, the model is able to better perceive features in regions proximal to the fixation location, and saccade to them (smaller saccades, lower variance). Moreover, the higher foveal magnification, enables analyzing the region of change with higher resolution, thereby leading to better performance. We show this systematic degradation in performance with increased saccade amplitude variance (Fig. 5F), with the underlying cause for each being the change in the foveal magnification factor. This provides a plausible mechanism for higher variance of saccade amplitudes in poor performers.

ii) No-change (negative) evidence threshold with decay: Second, we have incorporated a no-change threshold for breaking fixations in the model. Fixation intervals are no longer sampled from a Gaussian. Rather, if the accumulated evidence crosses a negative (no-change) threshold F_n_ (Table 1), the observer terminates the current fixation. We decayed the no-change threshold with different decay rates and studied its effect on model performance (see also response to Reviewer #1).

We show that a faster decay of the no-change bound, possibly reflecting a stronger “urgency” to break fixations, resulted in faster bound crossing and, therefore, shorter fixations, and directly impacts performance (Fig. 5D, upper inset). This provides a plausible mechanism for shorter fixation durations in poor performers.

iii) Prior odds ratio: Third, we have incorporated prior ratios into the likelihood-ratio based decision rule in the model (equation 2, revised manuscript). The prior odds ratio reflects an individual’s subjective belief in the prior probability of change to no change. With this modification, our formulation of an SPRT decision involves evaluating and integrating fully the logarithm of the Bayesian posterior odds ratio (product of the prior odds and likelihood ratio).

We demonstrate that a lower prior odds ratio (higher degree of belief in no change) biases the evidence accumulation process toward the negative threshold, and leads to shorter fixations, and impacts performance (Fig. 5D, lower inset). This provides a second, plausible mechanism for shorter fixation durations in poor performers.

We have also revised the text, based on these new modifications to the model, as follows:

**Figure 5**

We have revised our Results, when describing the model, as follows:

“Second, the observer integrates the “evidence” for change at location *A_i_*, by accumulating the logarithm of the likelihood ratio log(*L_i_(t)*), **along with the log of the prior odds ratio (*P_i_*),** as in the SPRT framework.

$E_{i}\left( t \right)=\left( 1-\gamma_{i}\left( t \right) \right)E_{i}\left( t-1 \right)+{log(L}_{i}\left( t \right))+\boldsymbol{log}\left( \boldsymbol{P}_{\boldsymbol{i}} \right)+W_{i}\left( t \right)$	… (2)

where *ɣ_i_* ∈[0,1] is a decay parameter for evidence accumulation at location *A_i_*, which simulates “leaky” evidence accumulation (15,29) with larger values of *ɣ_i_*, indicating greater “leak” in evidence accumulation**, *P_i_* is the prior odds ratio of change to no change (P(C)/P(N)) at each location**, *W_i_(t)* represents white noise*,* sampled from a uniform distribution (Table 1), to mimic noisy evidence accumulation (30). Here we assume that the prior ratio is constant across time and space, but nonetheless study the effect of varying prior ratios on model performance (next section). Both of these features – leak and noise in evidence accumulation – are routinely incorporated in models of human decision-making (29), and are grounded in experimental observations in brain regions implicated in decision-making (15). Evidence accumulation occurs in the original, physical space of the image, and not in the CVR transformed space (Fig. 4B). **Note that this formulation of an SPRT decision involves evaluating and integrating fully the logarithm of the Bayesian posterior odds ratio (product of the prior odds and likelihood ratio, *P_i_ × L_i_(t)*).**

Evidence accumulation is performed for each region in the image; $E_{i}(t)$ for each region is calculated independently of the other regions. If the accumulated evidence $E_{i}(t)$ crosses a positive threshold, *F_c_* (Table 1), the observer stops scanning the image and region *A_i_*, at which the threshold *F_c_* was crossed, is declared the “change region”. **If, on the other hand, the accumulated evidence crosses a negative (no-change) threshold *F_n_* (Table 1),** the observer terminates the current fixation and determines the next region to fixate, *A_k_*, based on a softmax probability function:

$p_{k} ={e^{\frac{E_{k}}{T}}/\Sigma}_{i=1}^{N}e^{\frac{E_{i}}{T}}$	… (3)

where *E_i_* is the evidence value for region *i*, *N* is the number of regions in the image, and T is a temperature parameter which controls the stochasticity of the saccade (decision) policy (Methods; see also next section). For selecting the next point of gaze fixation, we also matched directional saccadic biases typically observed in human data (31) (Fig. 4B, described in Methods section on “Comparison of model performance with human data**”). In some simulations we also decayed the no-change threshold (*F_n_*) with different decay rates (ζ; Table 1) and studied its effect on model performance. Because we observed virtually no false-alarms (signaling a no-change location as change) in our experimental data (0.06% of all trials; Methods) we did not model decay in the change threshold (*F_c_*), which would have yielded significantly more false alarms.”**

And when describing the effects of parameter variations in the Results, we have revised the text, as follows:

“Fourth, we tested the effect of varying mean fixation duration (μ_FD_) – a key parameter identified in this study as being predictive of success with change detection. The mean fixation duration is not a parameter of the model. We, therefore, varied the mean fixation duration, indirectly, by varying the prior odds ratio (*P*) and the decay rate (ζ) of the no-change threshold (*F_n_*). A lower prior odds ratio of change to no-change biases evidence accumulation toward the no-change threshold, leading to shorter fixations (and vice versa; Fig 5D, lower inset). On the other hand, a higher decay rate of the no-change threshold leads to a greater probability of bound crossing of the evidence in the negative direction, again leading to shorter fixations (and vice versa; Fig. 5D; upper inset). In either case, we found that decreasing (increasing) the mean fixation duration produced systematic deterioration (improvement) in the performance of the model (Fig. 5D). These results recapitulate trends in the human data, indicating that increased fixation duration may be a key gaze metric indicating change detection success.

Fifth, we tested the effect of varying the saccade amplitude variance ($\sigma_{SD}^{2}$) – the other key parameter we had identified as being predictive of change detection success. Again, because the variance of the saccade amplitude is not a parameter of the model, we varied this, indirectly, by varying the temperature (*T*) parameter in the softmax function: a higher temperature value leads to random sampling from many regions of the image, thereby increasing $\sigma_{SD}^{2}$ whereas a low temperature value leads to more deterministic sampling, thereby reducing $\sigma_{SD}^{2}$ (Fig. 5E, inset). With increasing saccade variance, performance dropped steeply (Fig. 5E). Finally, we also explored the effect of varying the foveal magnification factor (FMF) across a two-fold range. Saccade amplitude variance decreased robustly as the FMF increases (Fig. 5F, inset) (see Discussion). As with the previous simulation, we observed a systematic decrease in performance with increasing saccade amplitude variance, again, recapitulating trends in the human data.”

We have added a new paragraph in the revised Discussion, further elaborating on these results:

“Finally, the model provided mechanistic insights about key trends observed in our own experiments, specifically, a critical dependence of success rates on mean fixation durations and the variance of saccade amplitudes (Figs. 5D-F). Fixation durations in the model varied systematically either with altering the prior odds ratio or the decay rate of the no-change bound. Note that the prior odds ratio correspond to an individual’s prior belief in the prior probability of change to no change. The lower this ratio, the higher the degree of belief in no change, and the sooner the individual seeks to break each fixation. In our model, this was achieved by having the prior ratio biased evidence accumulation toward the no-change (negative) bound. Similarly, faster decay of the no-change bound, possibly reflecting a stronger “urgency” to break fixations, resulted in faster bound crossing and, therefore, shorter fixations.

Regardless of the mechanism, shorter fixation durations resulted in impaired change detection performance (Fig. 5E), providing a putative mechanistic link between fixation durations and change detection success in the experimental data. In addition, saccade amplitude variance modulated systematically with changes in the foveal magnification factor (FMF). We propose that this happens because with higher foveal magnification, the model is able to better perceive features in regions proximal to the fixation location, and saccade to them, thereby resulting in overall shorter saccades, and lower variance. Moreover, the higher foveal magnification, enables analyzing the region of change with higher resolution, thereby leading to better change detection performance. As a consequence performance degraded systematically with increased saccade amplitude variance (Fig. 5F), the common underlying cause for each being the change in the foveal magnification factor. This provides a plausible mechanism for higher variance of saccade amplitudes in “poor” performers.”

**III-b)** Next, the null models (lesions of the Bayesian SPRT rule starting on line 487) have no justification beyond that they are limits on the proposed model. There’s no discussion that the lesions to the model reflect plausible mechanisms. This is a necessary step to prove a functional stopping rule (such as the SPRT) is important, could an alternative stopping rule achieve the same results? Answering this question could provide mechanistic insight.

Based on the reviewer’s suggestion, we have performed the following additional analyses:

a) We have removed Control Model 2 where we randomly flip the log-likelihood ratio during evidence accumulation (±log L(t)). We agree with the reviewer that this control does not reflect neurobiological mechanisms.

b) We have added a new control model in which we implement a stopping rule based on the difference of the log posterior odds between successive timesteps. In this control model the stopping occurs not based on SPRT evidence accumulation, but rather based on *the derivative of this ratio* crossing a threshold. We show that this model also falls short of the SPRT model in terms of its match to human performance data. In the revised Results, we report:

“We compared the Bayesian SPRT search rule, as specified in our model, against three alternative control models with different search strategies or stopping rules: i) a model in which evidence decayed rapidly, so that the decision to signal change was based on the instantaneous posterior odds ratio alone; ii) a model in which the stopping rule was based on crossing a threshold “rate of change” of the posterior odds ratio, and iii) a model that employed a random search strategy (Methods). For each of these models, the average absolute difference in performance with the human data was significantly higher, compared with that of the original model (Fig. 6C; p<0.05 for 2/3 control models; Wilcoxon signed-rank test). Moreover, none of the control model’s success rates correlated significantly with human observers’ success rates (r=0.09-0.42, p>0.05, for all 3/3 control models; robust correlations).”

c) We repeated the simulations of the other two control models (rapid temporal decay of evidence, and model with random saccades) with the updated SPRT model, with the several new features outlined in this revision (response to III-a).

**Figure 6**

d) In the revised Discussion, we now discuss mechanisms that may underlie each of the three control models:

“We implemented three control models in this study. The first control model – in which evidence decayed rapidly (γ=1) – mimics the scenario of rapidly decaying short-term memory; this model signals the change based on threshold crossing of the instantaneous, rather than the accumulated, posterior odds. In the second control model, we employed an alternative stopping rule: a rapid, large change in the posterior odds ratio sufficed to signal the change. Such a “temporally local” stopping rule obviates the need for evidence accumulation (short-term memory) and may be implemented by neural circuits that act as temporal change detectors (differentiators). The third control model mimicked a random saccade strategy, with a high temperature parameter (T=10^4^) of the softmax function. This model establishes baseline (chance) levels of success, if an observer were to ignore model evidence and saccade randomly to different locations on each image, and arrive at the change region “by chance”. Each of these control models fell short of our SPRT model in terms of their match to human performance.”

We have also revised our Methods to reflect these changes:

“Next, we performed control analyses to compare the SPRT model with three other change detection models, each with particular differences in search strategy or stopping rule. First, we tested a model that failed to integrate evidence effectively by setting γ=1 in the evidence integration step (equation 2). Such a model completely ignores past evidence and makes decisions based solely on instantaneous posterior odds ratio (*L_i_(t)P_i_*). Second, we tested a model with an alternative stopping rule in which the change was detected based on the derivative of the posterior odds ratio (difference of log (*L_i_ P_i_*) between two successive timesteps) crossing a threshold. For these two models, threshold values for terminating the simulation were determined based on two pilot runs across all 20 images; thresholds were chosen such that the models provided a negligible proportion of false-alarm (<0.01%) comparable with our experimental data. Third, we tested a model in which evidence computation and accumulation were intact, but the model selected the next location of saccade with a random strategy. This was achieved by setting a high value of the temperature parameter (T) in the final softmax function (T=10^4^), which resulted in a nearly uniform probability, across the image, of selecting the next fixation (“random searcher”). For all three models, we identically matched the timing and distribution of fixation interval durations with our standard SPRT model. The distribution of absolute differences in performance between the human data and our model across images, and the corresponding distributions for control models were compared with paired signed rank tests (Fig. 6C).”

Finally, we have removed the previous claim in the Results that the Bayesian SPRT rule was “necessary for achieving this correspondence between human and model success rates” (deletions in lines 589-591, redlined version).

**III-c)** What I see right now is a validation of a novel model for predicting saccade patterns. That itself is important, and is worth leaning into. If that’s what the model is getting at, are there other datasets it can be compared against? This might be a big, time-consuming addition, but if the model is able to account for more experimental results than just those of the group, the impact of the model is greatly expanded.

Our model is relevant for predicting saccade patterns in *change blindness tasks.* Unfortunately, we could not find publicly available gaze datasets for change blindness tasks, which could be used to evaluate our model framework. On the other hand, this study introduces -- for the first time -- a substantial new experimental dataset on change blindness (n=39 subjects) with multiple high-resolution gaze metrics, collected *de novo.* Our dataset can be used to evaluate and compare our model with other change-detection gaze models in future studies. The new experimental dataset complements our novel model, and the fact that both are featured in the same study adds value to our findings.

**Minor Comments**

• Why use an SVM with a polynomial kernel? If the goal is feature importance and a nonlinear classifier, random forest (with Matlab’s Treebagger) with feature permutation would be more straightforward. It is less sensitive outliers and noninformative features. Furthermore, feature permutation as a measure of feature importance is a highly validated metric and relatively straightforward. I don’t anticipate the result would be different, but using that method could be easier for readers to understand and trust. It would probably only take an afternoon or so to implement in Treebagger, as you already have the processing pipeline for SVM in Matlab.

We have now performed this analysis and report the results in Figure 1D (revised manuscript). Treebagger selects features that are similar to those identified by the other feature selection approaches (Fig. 1D).

**Figure 1**

Revision to the Methods:

“Next, we sought to identify gaze metrics that best distinguished good from poor performers. For this we employed **four standard metrics** -- Fisher score (18), AUC change (19) and Information Gain (20) and **bag of decision trees (21)** – which quantify the relative importance of each feature for distinguishing the two groups of subjects (Fig. 1D). A detailed description of these metrics is provided next.

iv) Bag of decision trees is an approach for feature selection using bootstrap aggregation on an ensemble of decision trees. Rather than using a single decision tree this approach avoids overfitting by growing an ensemble of trees on independent bootstrap distributions drawn from the data. The most important features are selected by out-of-bag estimates of feature importance in the bagged decision trees. We used the *Treebagger* function, as implemented in Matlab, with saccade and fixation features as inputs to the model, which classified if the data belonged to a good or poor performer. The number of trees was set to 6, with all other hyperparameters set to their default values.”

• Figure 1B, cleanup the location of the dashed lines. Looks messy.

The lines have been replaced with inverted triangles (Fig.1B, above).

• Show the null distribution (shuffled labels) in the figure 1C, not just the chance level.

Shown with a violin plot (

Fig. 1C, above).

• Figure 3A, could be useful to show a subtraction of the matrices. Not necessary, but would clarify the point.

Subtraction of the matrices is now shown in SI Fig. S3A.

• Figure 4C/D Is there a way to visually connect the colors? I shouldn’t have to look to the legend to understand the connection.

We have used more distinctive colors and added numbers, to better make the connection between Figures 4C and 4D.

• In many figures (Figure 6 being the worst) the order of the panels is really confusing. They should be laid out sequentially.

We apologize for the confusion. This is now amended.

• Line 330 and line 331, “In other words,” is used twice.

Amended. Thank you.

• Please provide more numbering of equations in the methods section. That will make it easier when the paper is published for people to use it.

We have now numbered all key equations.
